# High-Efficiency Analytical Protein A Columns for High Sensitivity Monoclonal Antibody Titer Analysis

**DOI:** 10.1021/acs.analchem.5c04766

**Published:** 2025-11-18

**Authors:** Beatrice Muriithi, Fabrice Gritti, Martin Gilar, Yeliz Sarisozen, Matthew Lauber, Kevin Wyndham

**Affiliations:** 36565 Waters Corporation, 34 Maple Street, Milford, Massachusetts 01757, USA

## Abstract

The quantification of monoclonal antibodies (mAbs) using analytical Protein A affinity chromatography HPLC columns with porous particles of 20 μm or larger diameters is a common method in bioprocessing analytics. The limitations of this method are long run times, relatively broad peaks, and low sensitivity. To address this, we developed an analytical Protein A column packed with 3.5 μm nonporous particles in organosilica-modified hardware, designed to minimize nonspecific binding of mAbs and enable rapid, reproducible, and sensitive measurements. Experimental and theoretical results show that smaller, nonporous particles reduce analyte axial dispersion and mass transfer-related band broadening. This leads to three times narrower peaks and greater peak heights compared to those obtained with 20 μm fully porous particles. Although the column with 3.5 μm nonporous particles has more than ten times less surface area and 50% less Protein A density, its dynamic binding capacity is 62% at 10% breakthrough compared to a column of the same size packed with 20 μm porous particles. This implies that not all of the available surface area or ligand capacity is accessible for binding for the fully porous particles. Both columns demonstrated greater than 98% recovery and a low carryover of less than 0.3% for the analytical scale mass load. The use of 3.5 μm nonporous particles improves the speed, sensitivity, and efficiency of analytical Protein A chromatography, making it applicable to high-throughput bioprocess monitoring.

## Graphical abstract

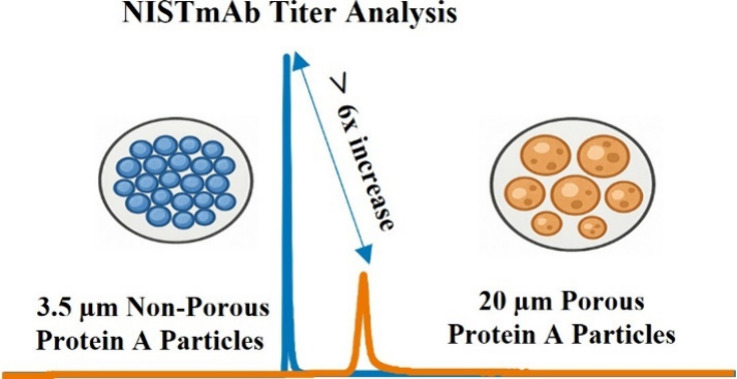

## Introduction

Affinity chromatography (AC) was first described in 1968 for the purification of enzymes. Other applications of AC were isolation, purification, and identification of low-abundance proteins,^1^ glycoproteins,^2,3^ histidine-tagged proteins,^4^ immunoglobulins,^5^ and mRNA.^6,7^ Affinity chromatography, in contrast to other chromatographic methods, utilizes highly specific interactions to retain the analyte of interest through the selectivity of an immobilized ligand (typically a protein or nucleic acid) that strongly interacts with the molecule of interest. The goal of AC is to enrich the target analyte (or class of analytes) while removing all other constituents present in the sample that have a negligible interaction with the sorbent-immobilized ligand. The analyte(s) retained on the column are then released with a (step) gradient of a mobile phase, after which the column is re-equilibrated with a binding mobile phase and prepared for the next sample load.^8^ AC essentially yields two fractions: a first peak containing the sample matrix and constituents that do not bind to the immobilized ligand and a second peak that contains the analyte of interest. AC is most often used for the purification of biomolecules, typically with a plastic (bioinert) or glass column housing, packed with particles of 50–100 μm, and operated in low-pressure mode with a mobile phase delivered by low-pressure LC instruments.^9^

One of the most common applications of AC is the isolation/purification of monoclonal antibodies (mAb). Immobilized Protein A or protein G sorbents, packed in a column format, are utilized to selectively bind to the Fc region of IgG antibodies in neutral pH buffers.^10^ The bound antibodies are subsequently eluted with suitable mobile phases. The AC technique is typically used for the small- to large-scale purification of therapeutic mAbs produced in bioreactors. There are several basic requirements for AC sorbents used for the isolation of therapeutic mAbs: (i) high dynamic binding capacity for mAbs, (ii) good sorbent stability and low bleed of immobilized affinity ligand, and (iii) high elution recovery and low mAb carryover. These requirements are achieved by using macroporous sorbents with high accessible surface area, using hydrophilic polymers or polymers modified with a hydrophilic layer to ensure low nonspecific interactions with sample proteins, and optimizing the covalent immobilization of the Protein A/G affinity ligand on the sorbent surface. AC columns are operated in various formats, including sorbent-packed beds, monoliths, and membranes sealed within a column housing. Each of these approaches has its benefits and limitations. The general method and formats of Protein AC were recently reviewed by Bilkova et al.^11^

Analytical AC columns are used for monitoring monoclonal antibody (mAb) production, quantifying mAbs, selecting clones, and optimizing bioprocess conditions. The requirements for the analytical application of affinity chromatography differ from those for preparative applications. Rather than the high binding capacity, an analytical application of Protein A should produce fast and reliable quantitative measurements, a wide calibration linear range, low carryover, and high sensitivity for low titers.^12,13^ Once optimized, an analytical Protein A column can be used for mAb characterization, for methionine-oxidation analysis,^14^ or for aggregate IgG measurement and host cell protein analysis.^15^

Analytical Protein A columns are designed to enable the sensitive quantification of IgG. To date, these AC columns have been packed with smaller particle-size (12–20 μm), yet still porous, particles. Alternatively, low-dispersion monoliths have also been used analytically.^16^ This type of column has a relatively small bed volume, internal diameter, and length, making it suitable for analytical measurements. Commercially available Protein A columns include POROS A 20 μm, 2.1 × 30 mm column (operational pressure limits of 2500 psi or 17 MPa); ProAqua Excel, 20 μm, 1000–2000 Å, 2.1 mm × 30 mm column (2500 psi or 20 MPa); BioMonolith Protein A, 4.95 × 5.2 mm column (2100 psi or15 MPa); Sartorius CIMac r-Protein A, 5.2 × 4.95 mm column (2175 psi or 15 MPa); and MAbPac Protein A LC, 4 × 35 mm, 12 μm nonporous, PEEK housing column (1000 psi or 7 MPa). While these devices permit routine IgG titer analysis, the particle size of the available Protein A columns is larger than those used in standard HPLC or UHPLC columns, and the column housings are rated for relatively low pressure. This limits the achievable peak volumes, analysis sensitivity, and attainable speed of analysis.

The goal of our research was to devise a protein A analytical column that offers high sensitivity, low peak volumes, high reproducibility, and rapid analysis of mAb titers. We chose a strategy that employs a high-pressure-capable, small particle size, nonporous sorbent with covalently immobilized protein A. The 3.5 μm protein A particles were packed into organosilica-modified column hardware designed to minimize mAb nonspecific binding and withstand operational pressures up to 15,000 psi.^17,18^ This column configuration enables users to utilize high mobile phase flow rates, allowing for fast analyses with a throughout of 30 to 60 analyses per hour. This study examines the theoretical performance of columns packed with smaller, nonporous particles compared to conventional, larger porous particles in analytical affinity chromatography and compares theoretical predictions with experimental data. We demonstrate that the high-efficiency Protein A column provides benefits to the performance of mAb titer assays.

## Experimental Section

### Materials and Reagents

The NIST monoclonal antibody (NISTmAb, a recombinant humanized IgG1k expressed in murine suspension culture) reference material (RM 8671) was purchased from NIST (Gaithersburg, MD), stored at −80 °C, and thawed as needed for use. Rabbit IgG (all subclasses of IgG 1,2,3,4) affinity purified (SKU IRBIGGAP) was purchased from Innovative Research (Novi, MI), stored at −80 °C, and defrosted when ready to use. Sodium phosphate monobasic (CAS 10049–21–5) and sodium phosphate dibasic heptahydrate (CAS 7782–85–6) were both purchased from Fisher Scientific (Hampton, NH). Sodium chloride (CAS.7647–14–5) and thiourea (CAS. 62–56–6) were purchased from Sigma-Aldrich (St. Louis, MO). Phosphoric acid (38%, CAS 7664–38–2) was purchased from Thermo Fisher (Waltham, MA). POROS A20 Protein A Affinity Resin (Catalog No. 1502903) was purchased from Thermo Fisher Scientific (Waltham, MA) and packed in Waters MaxPeak Premier Column hardware 2.1 × 20 mm from Waters Corporation (Milford, MA). POROS A20 20 μm, 2.1 mm × 30 mm column (Catalog No. 2100100) was purchased from Thermo Fisher Scientific (Waltham, MA). The Waters BioResolve Protein A Affinity Columns were prepared with 3.5 μm Protein A conjugated particles packed in 2.1 × 20 mm MaxPeak Premier Column hardware (SKU 186011369, Waters Corporation, Milford, MA). The 3.5 μm nonporous polystyrene/DVB particles were manufactured, hydrophilic-coated, and then conjugated with recombinant Protein A by Waters R&D and manufacturing teams (Taunton, MA). The specific surface areas (SSA) were measured using the multipoint N_2_ sorption method (Micromeritics ASAP 2400; Micromeritics Instruments Inc., Norcross, Ga). The Micro BCA assay kit (CAS 23235) was purchased from Thermo Fisher Scientific (Waltham, MA) and used to determine Protein A surface coverage. All reagents were used as purchased, without further purification. Buffers were prepared with Milli-Q water and filtered through a 0.2 μm filter (Cat. No. FB12566508) purchased from Fisher Scientific (Hampton, NH).

### Protein A Column Affinity Methods

NISTmAb samples were first prepared by diluting the original reagent (10 μg/μL) with freshly prepared 0.1 M sodium phosphate binding buffer (pH 7.4) to obtain concentrations of 1 and 0.1 μg/μL for calibration and detection limit studies. After vortexing, the samples were aliquoted into QuanRecovery Vials (Cat. 186009186, Waters Corporation, Milford, MA) and used immediately to minimize loss and degradation. Next, Protein A affinity columns were evaluated by using these samples as the model analyte. Columns were equilibrated with freshly prepared 0.1 M sodium phosphate binding buffer (pH 7.4) at 0.5 mL/min for 10 min before injection. NISTmAb samples were injected in triplicate at volumes from 0.1 to 10 μL, corresponding to protein loads of 0.01 to 10 μg. Chromatographic separations were performed on an ACQUITY Premier System equipped with a flow-through needle sample manager at 10 °C, a column manager at ambient temperature, and a TUV analytical UV flow cell. Binding was performed using 0.1 M sodium phosphate buffer (pH 7.4), and elution was achieved with 0.024 M phosphoric acid, pH 1.93. Affinity gradient methods were performed at 25 °C and a flow rate of 1 mL/min, consisting of 0.5 min of binding buffer, 1 min of elution, and 1.5 min of equilibration per run. The method steps were repeated twice to assess carryover, resulting in a total run time of 5 min. Effluent was monitored at 80 points/s at UV280 nm, and data was processed using Empower Software (Waters Corporation, Milford, MA). Quantitative analyses were based on the average peak areas from three injections.

To investigate how different dispersion mechanisms affect Protein A column performance, experiments were conducted under nonbinding conditions. We injected 1 μg of NISTmAb or 2 μg of thiourea and used 0.024 M phosphoric acid as the mobile phase for 1 min at various flow rates (0.1–1.0 mL/min). Columns were equilibrated with the mobile phase before each injection. All experiments were performed at a constant temperature of 25 °C to minimize the thermal effects on diffusion. The first and second moments were determined using Empower Software. These were then used with the Van Deemter equation to calculate plate heights (H) for each condition.

The maximum binding capacity of both Protein A columns was determined by using a rabbit IgG sample. First, several injections of rabbit IgG at 5.26 μg/μL, each with a 5 μL injection volume, were made onto the Protein A column using Multiple Injections in a Single Experimental Run (MISER).^19–22^ The column effluent was monitored with a TUV detector at 280 nm throughout the injections. The process was stopped once the column was saturated or the breakthrough was above 30%. Next, three additional injections were performed using a low-volume union (Part No. ZU1XC) purchased from Valco Instruments Co., Inc. (Houston, TX). These injections used similar amounts of rabbit IgG to determine the 100% breakthrough. Finally, the resulting data were used to calculate the total amount of rabbit IgG bound to the Protein A column.

## Results and Discussion

### Consideration for Protein A Affinity Column Design: Column Length, Particle Size, and Particle Porosity

We aim to develop a Protein A affinity method for the rapid and sensitive quantification of IgG. Based on chromatographic theory, we anticipate that this goal can be achieved with short columns packed with small, non-porous particles (e.g., 2.1 × 20 mm, 3.5 μm) rather than with long columns packed with particles larger than 10 μm. This rationale is supported by chromatographic theory: (i) short columns packed with small particle size have low dispersion and (ii) nonporous particles eliminate the diffusion in pores, providing for improvements in peak widths. The drawback of non-porous sorbents is their low surface area, which can lead to sample overloading and peak broadening. Very short columns, which operate at high flow rates (required for fast analyses), can also suffer from sample breakthrough, particularly for slowly diffusing macromolecules that may not be adsorbed on the column due to kinetic reasons.

To understand the limitations of AC, we initially evaluated the performance of short columns packed with a 3.5 μm nonporous sorbent versus 20 μm porous sorbents in silico. We calculated the surface area available in a 2.1 × 20 mm column packed with nonporous 3.5 and 20 μm porous particles (assuming an equal polymer skeleton density of 1.1 g/cm^3^, equal Protein A coverage, and interstitial particle porosity fraction ε = 40%, and identical static binding capacity). The SSA of the 3.5 μm nonporous particles and 20 μm porous particles was measured by low-temperature nitrogen adsorption at 1.53 and 20.9 m^2^/g, respectively (approximately 13.9 times more SSA with porous particles), suggesting that the porous sorbent should support greater on-column mass load. However, this is not expected to affect the peak width for a column packed with a nonporous sorbent when operated with an analytical mAb mass load.

Next, we investigated the residential time of the analyte on a 2.1 mm × 20 mm column at 1 mL/min. The column packed with 3.5 μm nonporous particles has a void volume V0 = 28 μL, while the column packed with 20 μm porous particles has volume *V*_0_ = 48.5 μL (assuming 50% particle porosity). The calculated elution time at 1 mL/min for unretained compound is 1.68 s (nonporous) or 2.91 s (porous sorbent). Probability of mAb (considering IgG1 molar mass 150 kDa) capture on column can be estimated from eq 1 (see Appendix 1) given by Crank^23^ using a simplification that the packed column can be described as a bundle of capillaries with straight and cylindrical flow channel of length *L* and *i.d.* corresponding to *d*_macropore_, which is related to particle size as defined by eq 2 (see Appendix 1).^24–27^ This scenario is schematically illustrated in Figure 1a, where slowly diffusing mAb molecules never encounter the Protein A sorbent (channel wall), which results in mAb partial breakthrough from the column. As mAb molecules migrate through a macropore channel, they may randomly collide with the Protein A-functionalized wall and get adsorbed. When we consider a scenario with a 2.1 mm × 20 mm column and a flow rate of 1 mL/min (upper limit of practical flow rate), the calculated capture probabilities were 93% (3.5 μm nonporous) and 63% (20 μm porous particles). However, for the convection contribution to the transverse dispersion of mAb sample across column^28^ (see Appendix 1), the calculated capture probabilities are 99% for 3.5 μm nonporous sorbent and 96% for the 20 μm porous sorbent-packed column.

**Figure 1:**
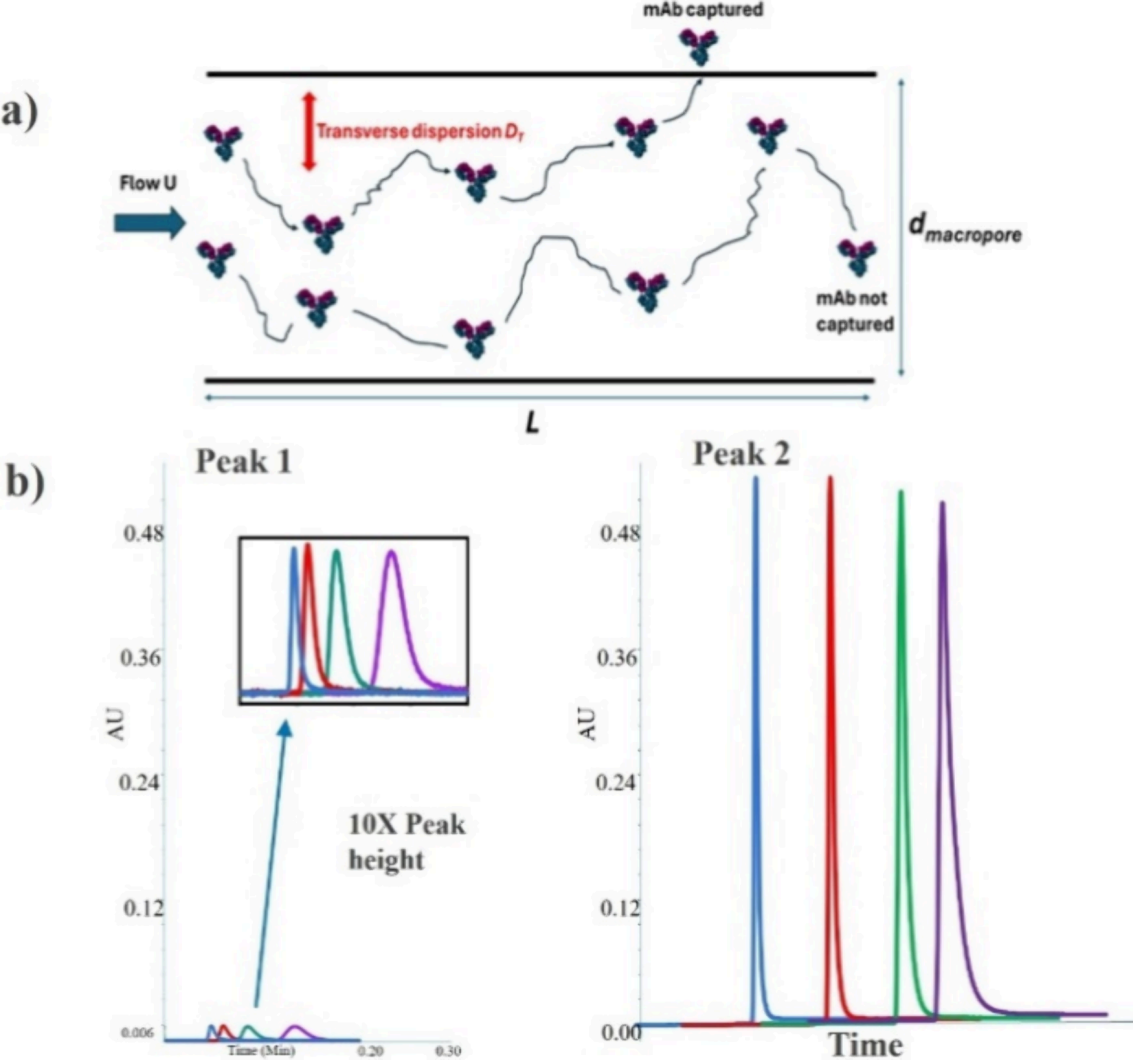
(a) Schematic of mAb transport in a cylindrical flow channel (length *L* and i.d. *d*_macropore_), and average velocity *u.* The transverse dispersion coefficient, *D*_*T*_, is defined in eq 1. In the case of unobstructed flow (open tubes), *D*_*T*_ equals *D*_*m*_, which is the bulk diffusion coefficient of mAb. (b) Peak 1 (breakthrough) and peak 2 (analyte elution) profiles for 1 μg of NISTmAb using a 3.5 μm nonporous column at various flow rates, 0.3 mL/min (purple), 0.5 mL/min (green), 0.75 mL/min (red), and 1 mL/min (blue). Inset: 10× zoom of breakthrough peaks, showing consistent ∼3.5% breakthrough across flow rates. The mAb elution peaks were captured within 0.2 min and were staggered manually. Data obtained with columns packed with 20 μm porous particles is shown in Figure S3.

We experimentally performed the Protein A method for both columns under similar conditions with sample loads of 1 μg of NISTmAb and at 0.3, 0.5, 0.75, and 1 mL/min. The chromatograms for columns packed with 3.5 μm nonporous particles are shown in Figure 1b; results for 20 μm porous particles are shown in Figure S3. With both columns, we did not observe any breakthrough; the peak area of peak 1 was constant at a level of ∼3.5% of the injected mAb sample, regardless of the sample concentration (0.5–5g/L mAb mass load). We believe that this peak corresponds to sample components with no affinity to Protein A impurity; for standards, this could be stabilizers or other excipient present in the mAb sample (observed independently in the size exclusion chromatography experiment; data not shown), while for a bioreactor sample, this peak corresponds to cell media components with no affinity to Protein A. We tested the specificity of columns packed with 3.5 μm nonporous Protein A particles using a fast 1.2 min method (Figure S2). Peak 1 corresponds to media components with no Protein A affinity, showing clear separation from the eluted NISTmAb and recoveries >98%. This demonstrates that such columns support faster runs, reducing instrument time, reagent use, and energy consumption, though the total method time is limited by LC system gradient delay. On low-dispersion systems like Acquity, injection-to-injection time is ∼2 min. Both experimental and in silico data suggest that short columns can be successfully used for measuring mAb titers.

Equation 1 (Appendix 1) was used to estimate the sample breakthrough at elevated flow rates within the range of 0.1 to 10 mL/min. The probability of mAb capture decreases modestly from 99.1% to 97.8% in the investigated flow rate range.

### Effects of Intraparticle Diffusivity

We estimated that the static binding capacity of the Protein A column packed with 20 μm porous particles is 13.9 times greater than that of 3.5 μm nonporous particles, based on available surface area. However, the dynamic binding capacity of the Protein A column using a porous sorbent depends on the experimental flow rate. Since mAb molecules require time to diffuse to the center of the porous particle, the analyte may not reach the entire particle, thereby reducing the binding capacity. The concentration profile of the mAb sample in the center of 20 μm porous (1000 Å) particles over time was simulated as shown in Figure 2, following Gritti and Meyyappan.^29^ The injected bulk mAb concentration was *c*_0_ = 5.26 μg/μL, and the flow rate was 0.1 mL/min. According to Figure 2, it takes a measurable time for the bulk concentration of the mAb sample at the center of the particle to normalize to 1.^29^ Only after 2 s does the external bulk mAb fully equilibrate with the particle center. At higher flow rates, the sample zone moves through the column quickly and the mAb molecules do not fully equilibrate at the particle center. Thus, only a “skin” of large porous particles is used for sample adsorption, so the column’s dynamic binding capacity is lower than its static binding capacity. In contrast, for nonporous particles, dynamic and static binding capacities are comparable even at fast chromatographic flow rates.

**Figure 2:**
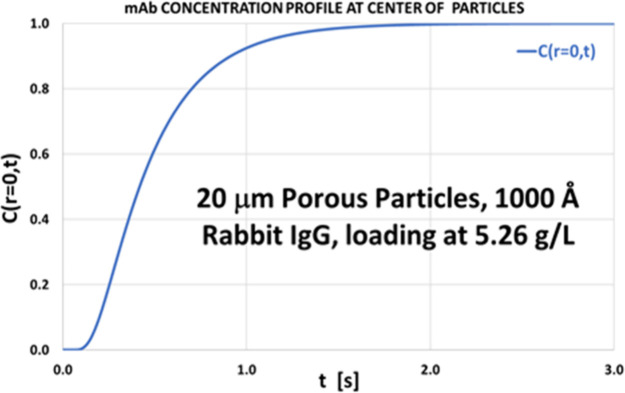
Simulation of mAb concentration profile in a 2.1 × 20 mm column packed with 20 μm porous particles exposed to 5.26 μg/μL rabbit IgG at 0.1 mL/min. Full equilibrium is reached after ∼2 s. Nonporous particles equilibrate faster (data not shown).

### Effects of Intraparticle Diffusivity on Sample Peak Width in the Elution

The slow mass transfer of the sample in the porous particles has an impact on the peak width during sample elution. Larger particle sizes will increase axial dispersion at a practical flow rate due to mass transfer resistance and slow intraparticle diffusivity *D_p_* (estimated at *D_p_* = 1.7 × 10^–7^ cm^2^/s for a 1000 Å pore size^30^) of the mAb across the particle diameter. For the empirical column plate height HETP analysis, see the Appendix, eq 3. Figure 3 shows the experimentally measured Van Deemter plots for 2.1 mm × 20 mm Protein A columns (plate height corrected for system dispersion) using thiourea and NISTmAb. The thiourea with fast diffusion shows consistently lower plate heights compared to the mAb on both columns. As anticipated, the A term is greater for larger 20 μm particles, adding to the plate height. The curve for nonporous particles remains flat, indicating zero *C* term contribution, while a linear increase in plate height with flow rate is observed for porous particles (thiourea results). The distortion of conventional Van Deemter plots is observed for mAb data. At high linear velocities, the curve for the 20 μm porous column flattens (contribution of *C* term decreases). This is an indirect sign of the effect discussed in Figure 2. At high analyte linear velocity, the mAb no longer has time to diffuse into the sorbent pores, causing the 20 μm porous particles to behave as nonporous ones, albeit with higher plate heights than columns containing 3.5 μm nonporous particles. This observation suggests that in preparative applications, columns packed with porous Protein A particles should be performed at relatively low flow rates. Figure 3 indicates that 3.5 μm nonporous particles will provide for narrower peaks and higher analytical sensitivity than columns packed with 20 μm porous particles.

**Figure 3:**
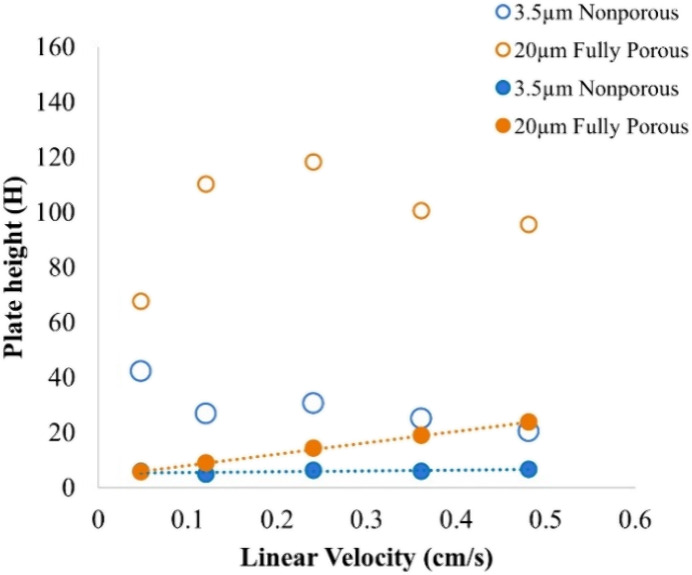
Van Deemter plots for Protein A columns (2.1 × 20 mm) packed with 20 μm porous (orange) or 3.5 μm nonporous particles (blue). Solid points correspond to data generated with thiourea and open circles correspond to data generated with NISTmAb. Smaller nonporous particles yield flatter curves and better efficiency. At high flow rates, 20 μm porous particles behave like nonporous due to limited pore access by NiSTmAb.

### Analytical Sensitivity of Protein A Affinity Columns

Figure 4 compares experimental chromatographic data for 2.1 × 20 mm columns packed with either 3.5 μm nonporous or 20 μm porous particles, using the same mass of mAb injected on the column. The mAb was loaded onto a column equilibrated in 100 mM sodium phosphate buffer, pH 7.5, and eluted with a gradient of 0.024M phosphoric acid, as described in the experimental section. The full chromatograms are shown in Figure S1. The column packed with 3.5 μm nonporous particles shows approximately 3-fold greater sensitivity, as demonstrated by the higher peak heights for a 1.0 μg sample load in Figure 4a. Across multiple on-column sample loads (Figure 4c,d), it also yields ∼3-fold steeper calibration curve slopes compared with the column packed with 20 μm porous particles. Peak widths were ∼0.3 s (4σ) vs ∼1 s, and peak volumes were 5–6 μL vs 17–20 μL for columns packed with 3.5 and 20 μm particles, respectively. Figure 4c,d shows overlaid chromatograms of NISTmAb loads ranging from 0.01 to 2.5 μg. The 2.5 μg sample load was the highest on-column load for a column packed with 3.5 μm nonporous particles, before the UV 280 nm signal was saturated and calibration curve nonlinearity was observed.

**Figure 4:**
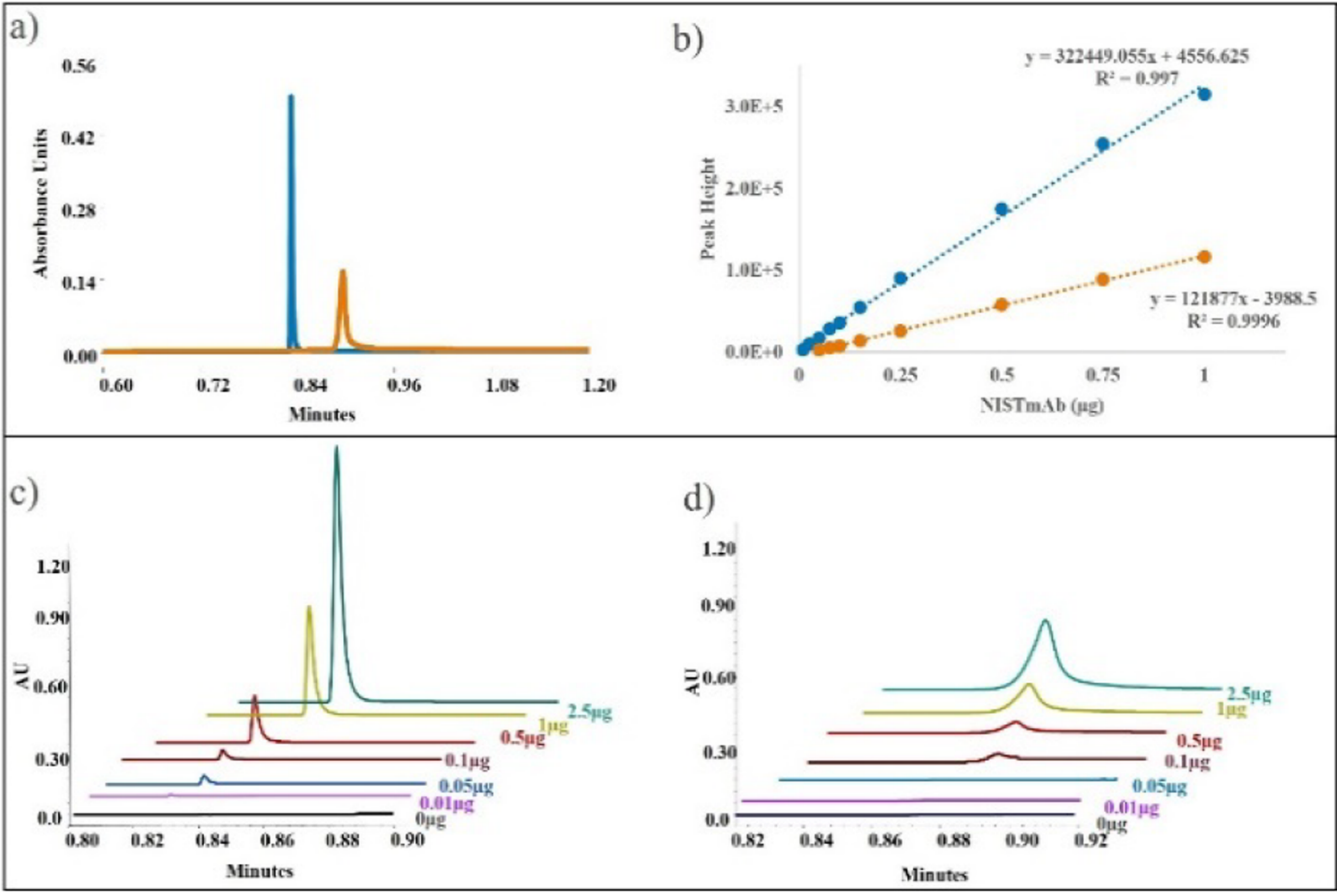
(a) Peak shape and sensitivity comparison between Protein A columns packed with 3.5 μm nonporous (blue) and 20 μm porous particles (orange) using 1 μg of NISTmAb as a sample. (b) Calibration curves were generated for NIST mAb sample amounts of 0.01–1 μg. Data are averaged over three injections, with the column packed with 3.5 μm nonporous particles (blue) and 20 μm porous particles (orange). (c,d) Representative chromatograms for Protein A columns packed with (c) 3.5 μm nonporous and (d) 20 μm porous particles.

Columns packed with 3.5 μm nonporous particles deliver sharper peaks, improving LOD and peak integration accuracy. This enables the detection of as low as 0.01 μg of mAb, compared to 0.05 μg with the column packed with 20 μm porous particles. Furthermore, sharper peaks improve the reliability of peak area integration, reducing data analysis and review time, especially for low-concentration samples. These columns are particularly advantageous in scenarios where sensitivity is paramount, such as low-abundance analyte detection, early-stage bioprocess development, such as in clone selection, and quality control of high-value biologics, applications requiring minimal sample consumption and higher throughput.

Figure 5 compares Protein A columns (2.1 × 20 mm MaxPeak Premier) packed with 3.5 μm nonporous and 20 μm porous particles for peak detection. The 3.5 μm column demonstrated higher sensitivity and improved LOD. NaCl-based elution protocols reduced sensitivity for both columns, likely due to changes in the UV280 nm detector response under high-salt conditions. Compared to POROS A20 columns (2.1 × 30 mm), the 3.5 μm column achieved over six times greater peak height and a 10-fold improvement in LOD (LOD-3.3σ /calibration slope)^31^ for 0.01–0.1 μg of mAb (see Figure S4).

**Figure 5:**
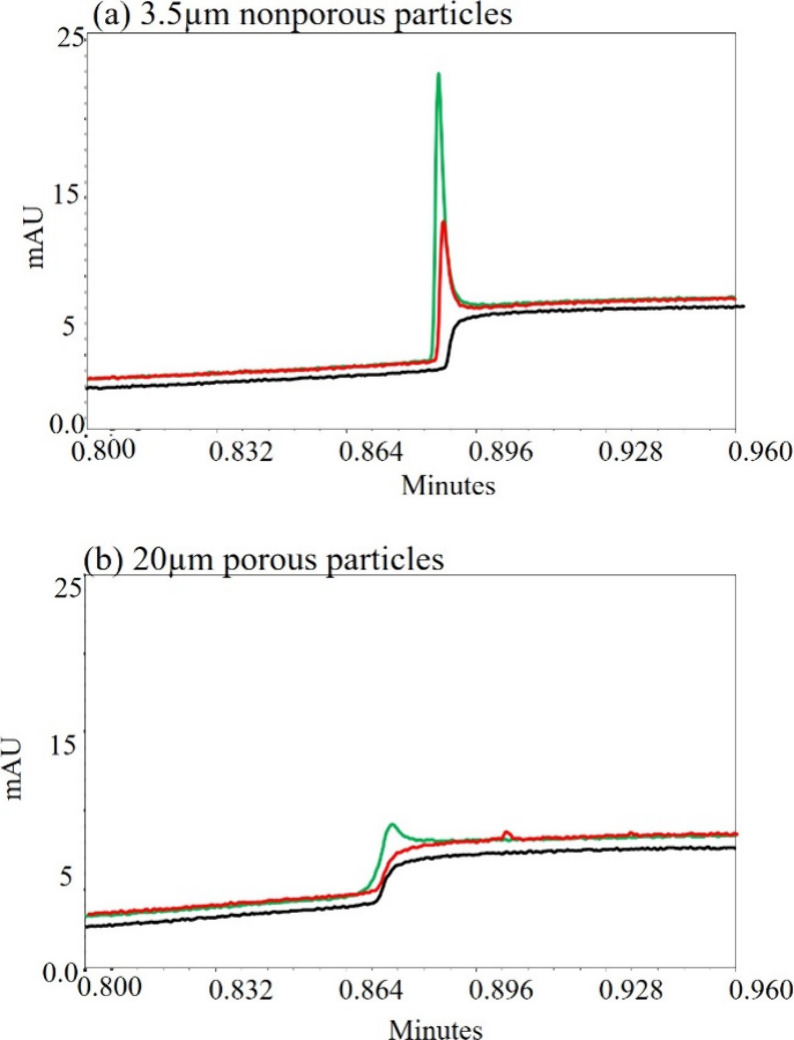
Overlays of blank (black), 0.01 μg (red), and 0.05 μg (green) of NISTmAb amounts on Protein A columns with 3.5 μm nonporous and 20 μm porous particles. The column packed with 3.5 μm nonporous particles offers enhanced sensitivity and peak clarity, improving the LOD and integration accuracy.

### Static Binding Capacity and Dynamic Binding Capacity of Protein A Columns

Static binding capacity is the maximum amount of mAb a Protein A column can retain under static conditions. For analytical Protein A columns (2.1 × 20 mm), a high capacity is not required for accurate titer measurements. While these columns may also be used to estimate parameters relevant to purification, their static binding capacity depends on the specific surface area of the Protein A particles, ligand coverage, and the accessibility of mesopores to mAb molecules. Although a 20 μm porous column offers approximately 14 times greater capacity (based on difference in specific surface area (SSA)) than a 3.5 μm nonporous column, both accommodate standard injection volumes (0.1–250 μL) and mAb amounts (1 ng-20 μg) within UV280 detection limits. Sensitivity, rather than capacity, is most important for reliable quantification in analytical workflows. Therefore, high-capacity sorbents are not necessary for titer determination in 2.1 × 20 mm analytical Protein A columns.

We utilized the MISER method^22^ to assess the maximum amount of monoclonal antibody (mAb) for a Protein A column with repeated injection of 5 μL of abbit IgG (5.26 μg/μL) at 0.1 mL/min. The MISER technique is an alternative to the dynamic binding capacity (DBC) method, typically used to determine binding capacity, which involves the use of a large single injection of a diluted sample to estimate column saturation with the sample, as demonstrated by the flow-through sample in breakthrough.^32,33^ The DBC technique is time-consuming and requires a high amount of valuable sample. In contrast, MISER streamlines the process by using multiple injections of equal concentrations and volumes in regular intervals. The dynamic binding capacity is defined similarly to the DBC method, which is detected as breakthrough, commonly defined as 10% of the injected sample. Breakthrough was normalized to the 100% peak area of the IgG sample injected without a Protein A column (the injector was connected to the UV detector via a void volume union). Figure 6 shows the results for both Protein A columns; each data point represents a single injection. The dashed line indicates the signal level of the 10% sample breakthrough through the column.

**Figure 6:**
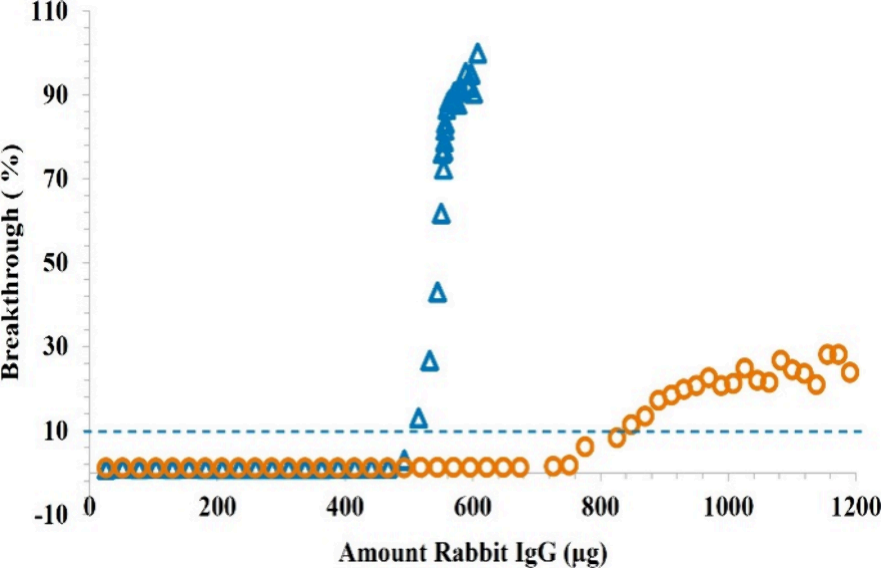
Comparison of dynamic binding capacity of 2.1 × 20 mm Protein A columns packed 3.5 μm nonporous particles (blue) and 20 μm porous (orange) particles. At 10% breakthrough, the column packed with 3.5 μm nonporous column bound 514 μg of IgG, only 38% less than the 20 μm porous column despite having lower surface area and ligand density.

Both columns show ∼1% signal from nonbinding impurities. The 3.5 μm nonporous column reached saturation quickly, with a DBC of 514 μg (62% of the 823 μg from the column packed with 20 μm porous). A slower breakthrough in the column packed with porous particles suggests that mass transfer limitations exist within the particles. Loading was stopped at 30% breakthrough for the column packed with 20 μm porous particles to minimize waste of the valuable sample. Short columns packed with small nonporous particles are ideal for analytical titer analysis and high-sensitivity applications. The column format can be scaled for purification needed in downstream characterization. However, their limited capacity makes them less suitable for large-scale batch purification.

## Conclusions

This study demonstrates that short Protein A columns packed with 3.5 μm nonporous particles significantly enhance analytical performance over traditional 20 μm porous columns. Despite having a lower surface area, the new column maintained 62% of the dynamic binding capacity, with sharper peaks (a 7-fold increase in peak height), higher sensitivity (>3 times), and smaller peak volumes (5–6 μL vs 17–20 μL). These improvements stem from reduced axial dispersion and mass transfer resistance. The small peak volumes obtained with a novel column are comparable to injection volumes of current chromatographic methods, such as size exclusion, which is useful for hyphenation for advanced mAb analysis. Sharper peaks also improve integration accuracy and reduce data analysis time, especially at low titers or in cases where sensitivity is crucial, such as in the early days of a bioreactor or during clone selection. Furthermore, short columns packed with nonporous Protein A particles enable faster runs—though limited by LC system delays and offer potential cost savings through reduced reagent use, instrument time, and energy consumption. The MISER method confirmed efficient binding performance. This platform is well-suited for high-sensitivity mAb titer analysis in bioprocess development, clone screening, and upstream process monitoring. These findings establish a mechanistic and quantitative framework for optimizing sorbents for analytical affinity columns, with a focus on achieving ultrahigh-performance liquid chromatography.

Waters, MaxPeak, BioResolve, ACQUITY, QuanRecovery, and Empower are trademarks of Waters Technologies Corporation. POROS is a trademark of Applies Biosystems, LLC. MAbPac is a trademark of ThermoFisher Scientific Inc. Milli-Q is a trademark of Merck KGaA. All other trademarks are the property of their respective owner.

## Supplementary Material



## Notes

Chromatograms of Protein A columns packed with particles of different size and porosity; specificity for short Protein A column; breakthrough and mAb elution, at various flow rates and sensitivity comparison of protein A columns, materials/methods for testing the specificity of Protein A columns packed with small nonporous particles, equations for binding and efficiency calculations of Protein A columns (PDF)
