# Supplementary material for: High-Efficiency Analytical Protein A Columns for High Sensitivity Monoclonal Antibody Titer Analysis
Source: Anal Chem. 2025 Nov 18;97(48):26349–56. doi: 10.1021/acs.analchem.5c04766 (PMC12874220; doi:10.1021/acs.analchem.5c04766)
Supplement: Supplementary file 1 [file ac5c04766_si_001.pdf]

Supporting Information

High Efficiency Analytical Protein A Columns for High Sensitivity Monoclonal Antibody Titer Analysis.

Beatrice Muriithi[1]*, Fabrice Gritti[1], Martin Gilar[1], Yeliz Sarisozen[1], Matthew Lauber[1], and Kevin Wyndham[1].

Affiliations : [1]Waters Corporation,

*Corresponding Author:

Email: beatrice_muriithi@waters.com

Table of Contents

a) Figure S1: Analytical Protein A column chromatogram…………………………....S3

b) Figure S2: Protein A column specificity and recoveries...…………………...……S3

c) Figure S3: Breakthrough and mAb elution peaks at different flow rates.……….....S4

d) Figure S4: Sensitivity comparison of Protein A columns.……………………..….S4

e) Materials/ Methods S1: Protocol for testing the specificity ………………………S4

f) Appendix 1: Equations used for calculations………………………………………S5

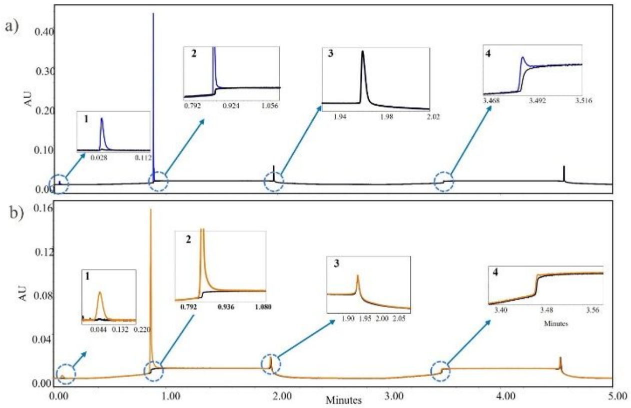

Figure S1: Chromatograms of a blank injection (black) and 1 µg NISTmAb (blue) on Protein A columns packed with (a) 3.5 µm nonporous and (b) 20 µm fully porous particles at 1 mL/min. Binding buffer: 0.1 M sodium phosphate (pH 7.4); elution buffer: 0.024 M phosphoric acid. Inset peaks numbered from 1 to 4 correspond to: (1) breakthrough, (2) mAb elution peak, (3) system peak due to column equilibration, and (4) carryover mAb peak position. Insets chromatograms are magnified for clarity. Mobile phase flow rate was 1 mL/min. The gradient was 0 to 0.5 min linear gradient from 100% A to 100% B, 0.5-1.5 hold at 100% B, 1.5 to 1.6 min linear gradient from 100% B to 100% A, 1.6 to 2.6 min hold at 100% A. The carryover was evaluated with the identical gradient (without injection of the sample) as follows: 2.6 to 3.1 min linear gradient from 100% A to 100% B, 3.1 to 4.1 min hold at 100% B, 4.1 to 4.2 min linear gradient from 100% B to 100% A and 4.2 to 5 min hold 100% A.

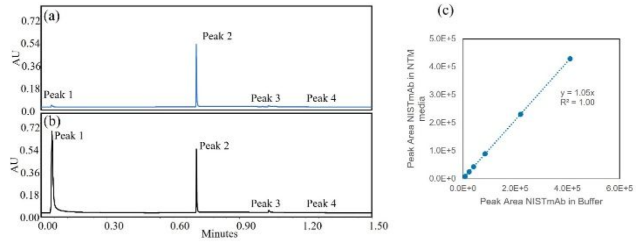

Figure S2: Protein A chromatograms of 1 µg NISTmAb in (a) buffer and (b) spiked into NTM CHO media, using columns packed with 3.5 µm nonporous particles at 1 mL/min. Binding buffer: 0.1 M sodium phosphate (pH 7.4); elution buffer: 0.024 M phosphoric acid. The step-gradient method: 0.1 min binding, 0.4 min elution, and 0.7 min re-equilibration (total 1.2 min). Peak1 for NISTmAb in buffer corresponds to components such as stabilizers or other excipients while in sample in media corresponds to A media component with no affinity to protein A, Peak 2 correspond to NISTmAb elution in both chromatograms, (3) system solvent switch, and (4) carryover. (c) Correlation of peak area for NISTmAb (0.1–5 µg, tested in triplicate) in buffer and NTM CHO media shows a correlation of 1, with a 4% difference attributed to 0.04 mg/mL Trastuzumab an impurity in the NTM media (also a mAb).

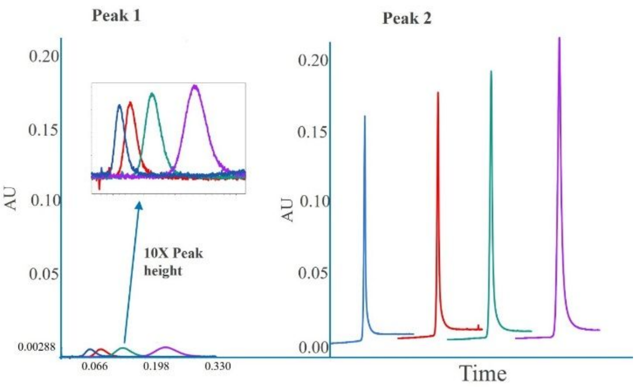

Figure S3: Illustration of breakthrough peaks (corresponding to non-binding species in the sample) and the elution peaks for 1µg NISTmAb sample analyzed with a column packed with 20µm fully porous particles. Data collected at different flow rates, where blue is 0.3mL/min, red is 0.5mL/min, green is 0.75mL/min, and purple is 1mL/min. The column had an average of 3.0% breakthrough by peak area, and carryover was observed at <0.3% irrespective of flow rate.

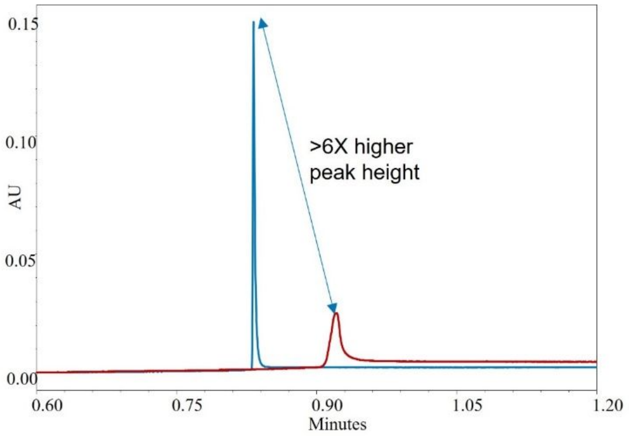

Figure S4: Shows a comparison of chromatographic peaks for Analytical Protein A 2.1x20mm columns packed with 3.5 µm non-porous particles (blue) and POROS A20 in PEEK 2.1x30 mm columns(red), performed under identical conditions as described in the experimental section using a 0.25µg NISTmAb sample load. Peak shape and peak height difference were observed, with 7.3 times higher peak height observed with Analytical Protein A columns packed with 3.5µm non-porous particles.

Materials and methods S1:

Clarified, non-transfected CHO media (NTM) with ~90% viability and 0.04 mg/mL Trastuzumab was obtained from Syd Labs (Hopkinton, MA), filtered through a 0.2 µm membrane, and stored at −80 °C be use and thawed as necessary. Spiked samples were prepared by adding NISTmAb stock (10 mg/mL) to NTM to yield 1 µg/µL. Varying injection volumes (0.1–10 µg on-column) were used to assess the specificity and recovery of Protein A columns

packed with 3.5 μm nonporous particles. Affinity chromatography was performed on an ACQUITY™ Premier System with a flow-through needle sample manager (10 °C), column manager at ambient temperature, and a TUV analytical UV flow cell. Affinity binding was carried out with 0.1 M sodium phosphate buffer (pH 7.4), and elution with 0.024 M phosphoric acid (pH 1.93) in a step gradient method at 25 °C and 1 mL/min flow rate: 0.1 min binding, 0.4 min elution, and 0.7 min equilibration per run. Samples were analyzed in triplicate.

Appendix 1

Affinity column can be with great simplification presented as a bundle of straight macropore channels. The probability of a molecule to be captured at the Protein A wall of a channel of internal diameter $d_{macropore}$ and length L is given by Crank[23] in Equation 1

$$P(d_{macropore,}L) = 2\int_{d_{macropore}}^{+\infty} \frac{1}{\sqrt{4\pi D_T t}} e^{-\frac{r^2}{4D_T t}} dr = erfc(z)\ ;z = \frac{d_{macropore}}{\sqrt{\frac{4D_T L}{u}}} \qquad \text{Eq. (1)}$$

where u is the average linear velocity, and D_T is the effective transverse dispersion coefficient across the channel. Where D_T=D_m for straight channels. In packed beds, D_T also involves a convective term, and D_T is than significantly larger than D_m as described.

$$d_{macropore} = 0.42\left(\frac{\varepsilon}{1-\varepsilon}\right)d_p \qquad \text{Eq. (2)}$$

Size of $d_{macropore}$ is related to the sorbent particle size d_p packed in the column. ε, the bed interstitial particle porosity fraction is assumed to be 40% of column volume. According to Eq. (2), $d_{macropore}$ =0.98 μm and 5.6 μm for the column packed with 3.5μm non-porous particles and with 20μm porous particles, respectively. The probability of capturing a mAb (bulk diffusion coefficient D_m= 4.0 x 10^{-7}cm^2/s) is assessed by Taylor dispersion analysis.

When we consider that convection contributes to the transverse dispersion of mAb across column [24] the effective transverse dispersion coefficient increased from 4.0×10^{-7} cm²/s to 1.2×10^{-5} cm²/s for 3.5 µm non-porous particles, and from 4.0×10^{-7} cm²/s to 4.3×10^{-5} cm²/s for 20 µm porous particles. This leads to an increased capture probability from 93% to 99% for smaller non-porous particles and from 63% to 96% for larger porous ones. [24-27]

For mAb and large Peclet numbers ($Pe=ud_p/D_m$), the empirical plate height equation reduces to:

$$HETP = A + \frac{W\mu}{1+Lu} + Cu \qquad \text{Eq. (3)}$$

Coefficient A accounts for the initial band width, axial bulk dispersion. *W* and *L* are empirical factors accounting for column wall effects and the outlet frit dispersion,[27] and C is the mass transfer resistance in the stationary phase. Note that C is equal to zero for non-porous particles
